# Validating a Coarse-Grained Potential Energy Function through Protein Loop Modelling

**DOI:** 10.1371/journal.pone.0065770

**Published:** 2013-06-18

**Authors:** James T. MacDonald, Lawrence A. Kelley, Paul S. Freemont

**Affiliations:** Division of Molecular Biosciences, Imperial College London, London, United Kingdom; Aberystwyth University, United Kingdom

## Abstract

Coarse-grained (CG) methods for sampling protein conformational space have the potential to increase computational efficiency by reducing the degrees of freedom. The gain in computational efficiency of CG methods often comes at the expense of non-protein like local conformational features. This could cause problems when transitioning to full atom models in a hierarchical framework. Here, a CG potential energy function was validated by applying it to the problem of loop prediction. A novel method to sample the conformational space of backbone atoms was benchmarked using a standard test set consisting of 351 distinct loops. This method used a sequence-independent CG potential energy function representing the protein using 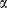-carbon positions only and sampling conformations with a Monte Carlo simulated annealing based protocol. Backbone atoms were added using a method previously described and then gradient minimised in the Rosetta force field. Despite the CG potential energy function being sequence-independent, the method performed similarly to methods that explicitly use either fragments of known protein backbones with similar sequences or residue-specific 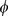/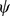-maps to restrict the search space. The method was also able to predict with sub-Angstrom accuracy two out of seven loops from recently solved crystal structures of proteins with low sequence and structure similarity to previously deposited structures in the PDB. The ability to sample realistic loop conformations directly from a potential energy function enables the incorporation of additional geometric restraints and the use of more advanced sampling methods in a way that is not possible to do easily with fragment replacement methods and also enable multi-scale simulations for protein design and protein structure prediction. These restraints could be derived from experimental data or could be design restraints in the case of computational protein design. C++ source code is available for download from http://www.sbg.bio.ic.ac.uk/phyre2/PD2/.

## Introduction

The prediction of protein structure to atomic level resolution and the design of de novo proteins with large scale backbone sampling are largely unsolved problems although there has been a great deal of progress in recent years. Both problems require the ability to rapidly sample a large number of backbone conformations. Sampling protein conformational space using full atom models can be prohibitively computationally expensive so a variety of different approaches have been developed to reduce the search space. This can be achieved by using coarse-grained (CG) protein models, by assembling backbone models from short fragments taken from known protein structures or by a combination of both of these methods.

Coarse-grained models have been increasingly used for modelling large biomolecules over long time scales due to the computational efficiency provided by these methods [1]–[3]. These models vary in the degree of coarse-graining with some models representing multiple amino acid residues with one interaction centre [4], some representing each amino acid residue with a small number of interaction centres [5]–[13], and others that are intermediate between minimal and full atom models [14]–[16]. Potential energy functions for CG models have been most commonly derived using statistics from from the Protein Data Bank (PDB) together with a suitable reference state [2]. Potential energy functions derived this way are known as knowledge-based or statistical potentials. It is also possible to derive CG potential energy functions from physical principles [17].

While CG models in the past were mostly used as toy models to study the general principles of protein folding [18], [19] they are now becoming sufficiently accurate and transferable to be used for more directly useful applications. For example, CG models are widely and successfully used in protein structure prediction methods with both lattice models [6], [8] and off-lattice methods [20]–[22]. CG models coupled with fragment replacement methods have been particularly successful. Backbone fragments are generally assembled in a Monte Carlo based procedure to assemble a new overall fold. As well as reducing the search space, these methods also have the advantage of guaranteeing models that have protein-like local conformational features. When these techniques are used for modelling loops, a loop closure method is required to ensure that the end of the loops connect the anchor residues in a geometrically correct way. Another disadvantage is that it is not easy to sample conformations using fragment replacement with additional restraints that could come from experimental information or for protein design applications. Fragment replacements are inherently non-local and highly disruptive moves so acceptance rates can be very low with additional restraints. It is also harder to use more advanced sampling techniques such as metadynamics [23] or umbrella sampling [24] as fragment replacement violates detailed balance in most common implementations [25] and this would be even more difficult when coupled with loop closure methods as is necessary in loop modelling. The ability to sample loop conformations with protein-like local structural features directly from a CG potential energy function could be one way of avoiding these problems.

The accuracy of full-atom reconstruction depends on the level of coarse-graining [16]. A number of methods have been developed to rapidly reconstruct mainchain atoms from C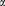 atoms [26]–[29]. Sidechains can then be added to the mainchain using fast rotamer-based methods [30], [31]. When transitioning between CG and full atom models it is important to retain good model structure quality. However, even in many full atom molecular mechanics force fields the modelling of backbone torsion angles has been problematic but recently efforts have been made to address this [32], [33]. A key feature of the C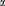 CG potential used in this study is its emphasis on protein-like local structure [11].

For most protein sequences, experimentally determined structures of homologous sequences are available and can be used as templates for accurate modelling [34], [35]. These homology models often have missing sections of the peptide chain where new residues have been inserted during the course of evolution. In these cases these loops will need to be predicted using *de novo* methods. Loop modelling is also important for computational protein design applications where the backbone structure needs to be redesigned in order to satify some functional constraints [36]–[38]. Loop modelling presents a rigorous and stringent test of *de novo* structure prediction methods due to their high degree of structural variability and a weaker sequence-structure relationship compared to secondary structure elements. While many loop prediction methods have been previously described [39]–[50], there is only one study on the use of C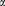 CG models for loop prediction without the use of backbone fragments from known protein structures [51].

In this paper we validate a previously developed sequence-independent CG potential energy function [11] by comparing its performance to some existing fragment and loop closure based methods. Full atom models are constructed from the sampled CG models, gradient minimised in a full atom potential energy function. The lowest energy structures were found to predict loop conformations surprisingly well with a high proportion of sub-Angstrom RMSD predictions.

## Results

The method presented here was compared to RAPPER [41] and FALCm4 [52] as the use of the same test set enables a direct comparison using the same metrics presented in those papers. RAPPER was taken as representative of methods that use a dihedral angle build-up method while FALCm4 was taken as representative of fragment replacement methods. The aim of this work was to determine whether it was possible to sample loops within the radius of convergence of full-atom refinement methods using a coarse-grained C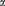 model.

### Loop Conformational Sampling

The loop prediction benchmark test proposed by Fiser *et al* [39] and filtered by DePristo *et al* [41] was used to assess the performance of the loop modelling methodology. This set contains loop targets of two to twelve residues in length. For each target, 4000 backbone loop conformations were sampled using a simulated annealing protocol (see Methods) using the potential energy function described in equation (1). As an additional control a further set of 4000 backbone loop conformations were sampled for each target where only the 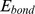 (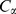 – 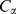 pseudo-bond term) and 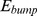 (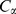 – 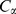 steric repulsive term) terms were included. This was carried out in order to determine the degree to which the other terms in the potential energy function were enhancing conformational sampling and is referred to as the “control” in the following text. The PD2 method introduced in this paper ensures that loops are always fully closed and the anchor residues are never moved. This is not always the case with the other loop sampling methods [50].

**Figure 1:**
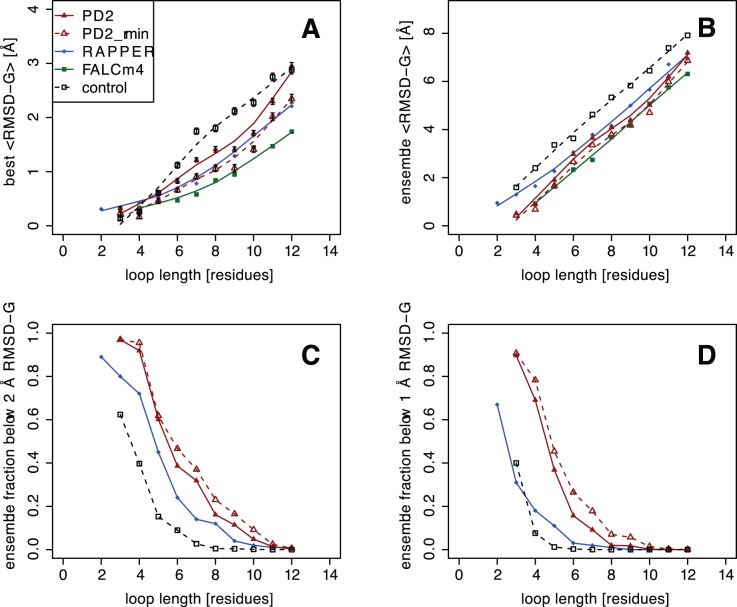
Loop sampling RMSDs by loop length. (A) mean lowest RMSD-G where the error bars represent 95% confidence intervals estimated by boostrapping. (B) Ensemble mean RMSD-G. Ensemble fraction (C) below 2 Angstrom and (D) 1 Angstrom RMSD-G. PD2 refers to loop decoys prior to minimisation in the Rosetta potential energy function, while PD2_rmin refers to loops decoys after Rosetta minimisation. Control refers to loops generated using a minimal C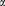 potential energy function (see text).

The RAPPER [41] and FALCm4 [52] methods were benchmarked using the same test set used in this paper. In both of these methods 1000 loop conformations were sampled rather than 4000 in this paper. In order to allow direct comparison with the results produced by RAPPER and FALCm4, 1000 loop conformations were resampled from the 4000 generated loops to estimate comparable statistics using the R statistical package “boot” to carry out a stratified bootstrap with 1000 replicates (Figure 1A). All RMSD-G values were calculated using the backbone heavy atoms N, CA, C, and O without superposition as defined by DePristo *et al* [41]. The best RMSD-G values were comparable to the RAPPER and FALCm4 methods and significantly better than the control (Figure 1A and Table S1 in File S1). Ensemble RMSD-G values were similar to FALCm4 but lower than for RAPPER (Figure 1B and Table S2 in File S1). Interestingly, a higher proportion of the PD2 loop ensemble lay below the 1 Å and 2 Å RMSD-G than both RAPPER and the control (Figure 1C and 1D, Tables S3 and S4 in File S1). This shows that near native loops were frequently sampled and could enhance the chance of selecting the correct conformation. At this stage no sequence information was incorporated into the PD2 loop sampling method but it still appeared competitive with methods that did include this information. RAPPER samples residue dependent discrete Ramachandran angles while FALCm4 is a fragment replacement-based method that selects fragments based on sequence similarity.

### All-atom Structure Refinement and Model Selection

**Figure 2:**
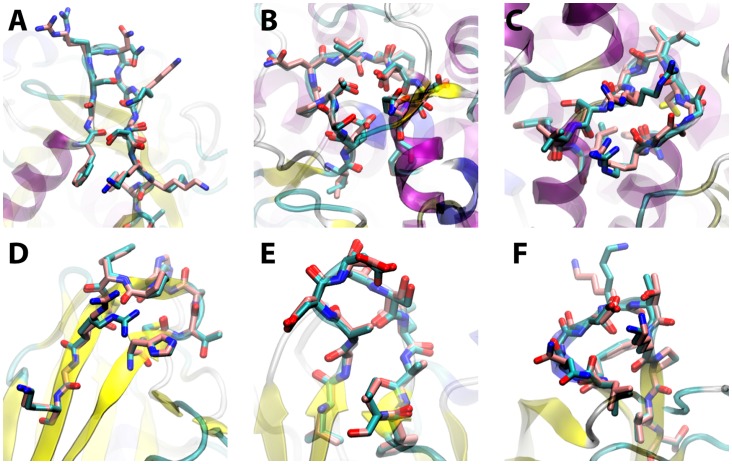
Example sub-Angstrom PD2_rmin loop predictions (pink) compared with the crystal structures (cyan), for loops of length (A) 12 (PDB: 2cpl 145–146), (B) 12 (PDB: 1arp 201–212), (C) 12 (PDB: 2pgd 361–372), (D) 11 (PDB: 1aaj 91–101), (E) 11 (PDB: 1plc 5–15) and (F) 11 (PDB: 1knb 521–511) residues.

**Figure 3:**
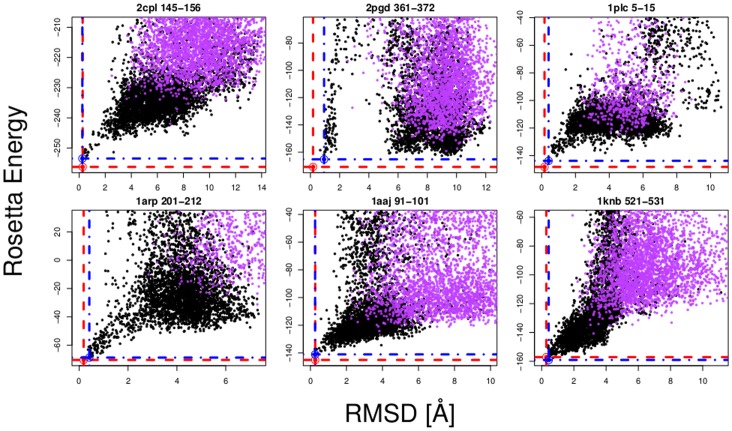
Backbone RMSD-G vs. Rosetta energy scatter plots for the loops shown in Figure 2. The red point indicates the energy minimised crystal structure loop and the blue point indicates the lowest energy PD2_rmin decoy loop conformation. The black points correspond to PD2_rmin loop decoys while the purple points correspond to the control_rmin loop decoys. Scatter plots for all loops are shown in File S1.

Sidechains were added to the generated backbone loops using the default Rosetta simulated annealing repacking algorithm and the whole loop (including the backbone) was then gradient minimised in a iterative manner as described in the Methods. The lowest energy loop was taken as the prediction. The results were comparable to existing methods and in some cases better (Table 1). Overall the method successfully predicted to sub-Angstrom accuracy 196 out of 351 loops in the test set (examples shown in Figures 2 and 3). In comparison, the control sampling method predicted 91 out of 351 loops in the test set to sub-Angstom accuracy and most of these were the short loops. Of the 174 loops of 8 residues or longer, 48 were predicted to sub-Angstom accuracy but none in the control. This indicates that while sequence independent coarse-grained statistical potential was significantly improving conformational sampling, the control method can successfully sample sub-Angstrom conformations only in the short loops where extensive search is possible. Previous studies have shown that exhaustive conformational searching taking into account crystal contacts together with a good all atom energy function can produce extremely good results [42]. However, this approach does not scale well, can take days of computational time to run and does not seem to work well on all loop test sets [50].

**Table 1:** Mean loop prediction accuracy for published methods and the method described in this work (PD2_rmin).

	backbone RMSD (Å)							
loop length	RAPPER	LOOPY	Rosetta	FALCm4	FREAD	CABS	PD2_rmin	control_rmin
2	0.35							
3	0.37						0.32	**0.23**
4	0.47			0.54	1.29	1.5	**0.30**	0.80
5	0.90	0.85		0.92	2.19	2.0	**0.68**	1.54
6	0.95	**0.92**		1.36	1.79	2.0	1.07	1.81
7	1.37	1.23		**1.17**	2.53	3.0	1.39	2.94
8	2.28	**1.45**	**1.45**	1.87	2.88	3.5	1.85	3.66
9	2.41	2.68		2.08	3.08	3.8	**2.01**	3.87
10	3.48	**2.21**		3.09	4.25	3.8	2.81	4.88
11	4.94	3.52	3.62	**3.43**	4.55	5.9	3.88	6.43
12	4.99	**3.42**		3.84	3.99	6.0	4.24	6.87

Values for RAPPER were taken from de Bakker *et al* [64], for LOOPY from Xiang *et al* [40], Rosetta from Rohl *et al* [43], FALCm4 from Lee *et al* [52], FREAD from Choi *et al* [50], CABS results were estimated from Figure 1 of Jamroz *et al* [51]. It must be noted that these results do not all come from the same test sets so are not directly comparable. Other methodological differences also make comparison difficult. For example, the Rosetta method repacks all sidechains. The results presented in this table for RAPPER, FALCm4, PD2_rmin and control_rmin are directly comparable as these are predictions based on the Fiser test set [39].

**Figure 4:**
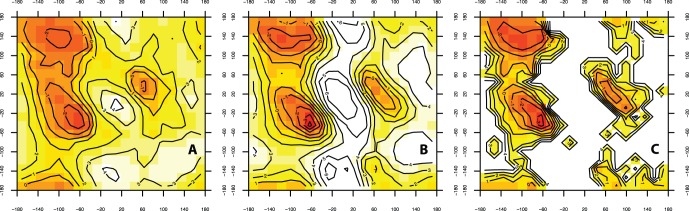
Ramachandran plot distribution of non-proline *trans*-conformation (A) PD2 decoy loop residues, (B) all SCOP40 residues, and (C) native loop conformations in the Fiser test set.

As a measure of backbone structure quality, the Ramachandran distribution was calculated for all generated loop decoys (Figure 4). Most features of the Ramachandran were reproduced in the loop decoys however there is still room for improvement. The dihedral angle distribution of the generated backbones is a function of both the C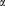 atom positions and of the method used to rebuild the mainchain atoms from the C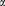 positions.

### Loops from Recently Deposited Structures

**Figure 5:**
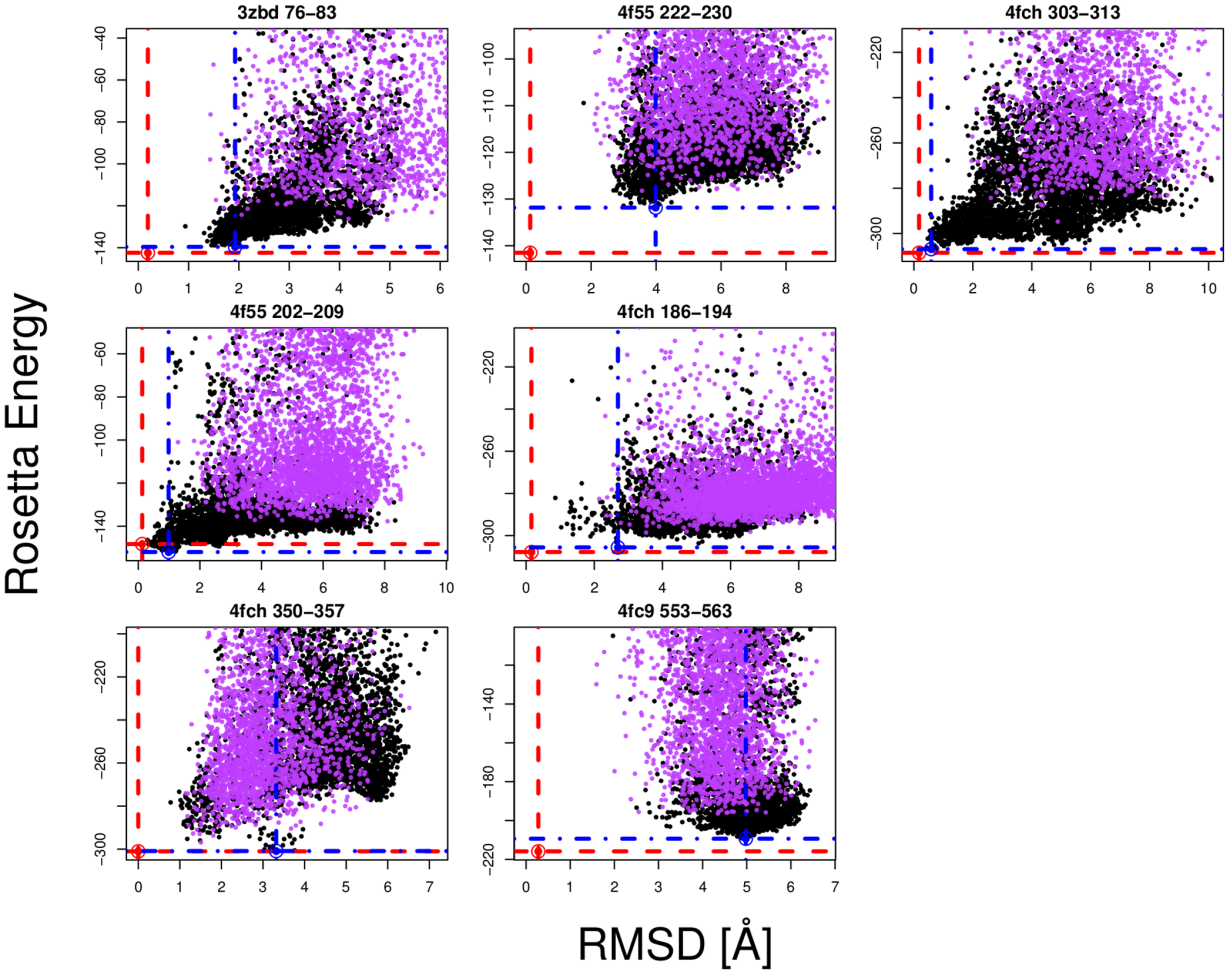
Backbone RMSD-G vs. Rosetta energy scatter plots for loops taken from a newly deposited set of structures (Table 2). The red point indicates the energy minimised crystal structure loop and the blue point indicates the lowest energy PD2_rmin decoy loop conformation. The black points correspond to PD2_rmin loop decoys while the purple points correspond to the control_rmin loop decoys.

Predictions were carried out on a new loop set taken from recently desposited structures with low sequence or structural similarity to solved structures deposited in the PDB before April 2012 (see Methods; Tables 2 and S5 in File S1; Figure 5). Of these seven loops, sub-Angstom conformations were sampled for 5 loops but no sub-Angstom conformations were sampled by the control. Two sub-Angstrom predictions were made but none were made for the control method. The CG potential energy function appears to be consistently sampling lower RMSD and lower energy loop conformations for both the original test set and the new test set.

**Table 2:** Loops predictions for newly deposited structures with low sequence and structure similarity to previous structures.

			lowest RMSD loop sampled (Å)				lowest energy loop RMSD (Å)	
PDB	residues	length	PD2	PD2_rmin	control	control_rmin	PD2_rmin	control_rmin
3zbd	76A–83A	8	1.23	**0.93**	1.69	1.43	**1.92 (−140)**	4.94 (−127)
4f55	202A–209A	8	0.86	**0.30**	1.48	1.29	**0.98 (**−**152)**	7.32 (−139)
4f55	222A–230A	9	2.84	1.77	1.94	**1.58**	3.99 **(**−**132)**	**3.57** (−128)
4fc9	553A–563A	11	2.65	1.97	1.94	**1.59**	4.98 **(**−**209)**	**4.35** (−196)
4fch	186A–194A	9	1.56	**0.86**	2.03	2.15	**2.70 (**−**306)**	3.52 (−299)
4fch	303A–313A	11	0.90	**0.45**	2.01	1.76	**0.59 (**−**307)**	4.88 (−295)
4fch	350A–357A	8	1.38	**0.78**	0.99	1.14	3.32 **(**−**301)**	**1.51** (−297)

Values in brackets are corresponding Rosetta energies.

## Discussion

We have shown that CG sampling techniques have the potential to be viable methods for atomic resolution loop prediction. This could be further improved with more advanced sampling techniques such as metadynamics [23] and incorporating sequence-dependent terms in the CG potential energy function. As loops are sampled from a potential energy function it would be possible to include extra restraints from experimental data or from contact prediction [53]. The CG potential energy function used in this work was initially developed as a method for the design of *de novo* backbone scaffolds [11]. The results of the paper confirms that it is sampling protein-like loop conformations more frequently than the control and that it works surprisingly well despite the sequence-independent nature of the energy function. It would be possible to incorporate functional geometric constraints as part of a computational protein design pipeline with large scale backbone sampling.

A high proportion of the sampled loop ensemble appears to be close to the native conformation (Figure 1 and Table S4 in File S1). This suggests that CG loops could be clustered prior to full-atom refinement in order to save time. It is also notable that the minimised native loop almost always has the lowest Rosetta energy (Figures S1 to S10 in File S1). This supports a previous observation that the main bottleneck in *de novo* protein structure prediction appears to be conformational sampling [54]. This work suggests that CG models of protein structures as part of a hierarchical approach can achieve atomic level accuracy.

## Materials and Methods

### C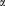 Potential Energy Function

The 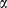-carbon potential energy function used a sub-set of terms from a previously described potential energy function [11] that is derived using a 27-“letter” structural alphabet [55]. This was composed of 5 terms (1).
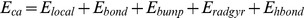
(1)


Where 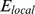 was composed of harmonic pseudo bond angle, dihedral terms and reference energies which vary as a function of their structural alphabet classification, 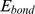 was a pseudo bond term, 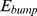 was a soft steric repulsive term, and 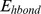 was a pseudo hydrogen bonding statistical potential term using pseudo N and O atoms as defined by Levitt [5]. The 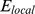 reference energy terms are parameterised such that the equilibrium distributions of each structural alphabet “letter” and each pair of consecutive “letters” reproduces that observed in the PDB (protein data bank) [11].

### C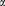 Monte Carlo Move Set

The move set consists of crankshaft moves (analogous to backbone backrub moves), bond moves where two 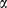-carbon atoms are moved by equal amounts in opposite directions along the bond vector and angle moves where two outside 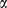-carbon atoms are rotated by equal and opposite amounts such that the bond angle is changed and the rotation axis is normal to the plane defined by the three 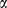-carbon atoms. All three of these move types are local moves that do not propagate along the whole chain.

### Backbone Potential Energy Function

A backbone potential energy function was used for conjugate gradient minimisation after rough backbone atom positions were added to 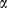-carbon models using a previously described method [27] in order to regularise the backbone stereochemistry. This included bond angle, bond length, torsion, improper torsion, 1–4 Lennard-Jones and 1–5 Lennard-Jones terms taken directly from the OPLS-UA force field [56], a soft steric repulsive term to prevent backbone clashes (as described in [11]) and reimplementation of the Rosetta backbone-backbone hydrogen bonding statistical potential [57].
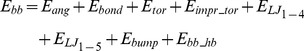
(2)


### Ensemble Generation

Initial loop 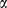-carbon positions were generated by linear interpolation between the fixed anchor 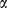-carbon with the addition of a small random vector displacement followed by conjugate gradient minimisation using only the 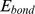 and 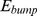 terms from (1). The initial positions were then relaxed in the full 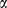-carbon potential using Monte Carlo simulated annealing for a total of 12000 steps. Conformations were generated by an inner cycle of 400 simulated annealing steps at the 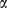-carbon level followed by the addition of initial backbone positions by a fast look-up method [27]. The annealing schedule consisted of 50 steps with 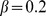, 100 steps with 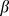 linearly increasing from 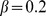 to 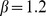 and finally 250 steps with 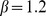 (where 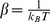). These conformations were accepted and then minimised in the backbone potential energy function if the number of residues in the loop with 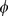/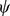 dihedral angles that lay in strictly forbidden regions of the non-residue specific Ramachandran plot, 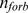, was 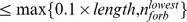, where 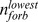 was the lowest previously accepted value of 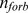. This was designed to prevent the algorithm getting stuck with no acceptable loops. At this stage 47% of generated loops were rejected.

### Gradient Minimisation and Selection with the Rosetta Energy Function

Sidechains were added using the default Rosetta simulated annealing repacking algorithm and the loop atoms gradient minimised in the Rosetta all atom potential energy function using a PyRosetta [58] script consisting of 15 outer cycles and 4 inner cycles. Each of the 4 inner cycles consisted of sidechain repacking followed by gradient minimisation. The weight of the repulsive term of the Lennard-Jones energy was gradually ramped up during the 4 inner cycles in the order 0.02, 0.25, 0.550 and finally 1.0. This was designed to replicate the Rosetta Fast Relax protocol [59]. The backbone and sidechains of the rest of the protein were kept fixed in their experimentally observed positions and the lowest energy structure generated during the protocol was retained. The lowest energy loop decoy was selected as the final prediction.

### Selection of New Loops from Recently Deposited Structures

Protein structures solved after April 2012 with novel folds were determined using a hierarchical approach based first on sequence similarity and then on structural similarity. First, the sequences of all structures solved after this date and greater than 20 amino acids in length (10,239) were BLASTed [60] against all PDB sequences deposited before this date. Any matches with a reported BLAST E-value 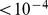 were removed as clear homologues leaving 1350 sequences. The corresponding structures of these 1350 sequences were then structurally compared to a representative set (pairwise sequence identity 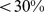) of the PDB taken from the PISCES [61] server with a date before April 2012 using MAMMOTH [62]. Any protein with a MAMMOTH hit with an E-value 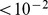 were discarded as structurally similar to an earlier deposited structure, leaving 361 proteins with potentially new folds. Many of these proteins were short (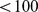 residues) suggesting that they may not constitute a genuine fold. After removing any structures 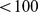 residues, this left 24 potential structures. Finally, of these 24 structures, any that were not high resolution crystal structures (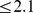 *Å*), contained chain breaks/missing residues, or had no loops in the range 8–12 were removed leaving a final set of 4 structures and 7 loops (Table 2). Loops were determined as contiguous sections of coil or turn as defined by STRIDE [63].

## Supporting Information

File S1 **Supporting figures and tables.**
(PDF)

## References

[1] Head-Gordon T (2003) Minimalist models for protein folding and design. Current Opinion in Structural Biology 13: 160–167. 12727508 10.1016/s0959-440x(03)00030-7

[2] Tozzini V (2005) Coarse-grained models for proteins. Current Opinion in Structural Biology 15: 144–150. 15837171 10.1016/j.sbi.2005.02.005

[3] Clementi C (2008) Coarse-grained models of protein folding: toy models or predictive tools? Current Opinion in Structural Biology 18: 10–15. 18160277 10.1016/j.sbi.2007.10.005

[4] Arkhipov A, Freddolino PL, Imada K, Namba K, Schulten K (2006) Coarse-grained molecular dynamics simulations of a rotating bacterial agellum. Biophysical Journal 91: 4589–4597. 16997871 10.1529/biophysj.106.093443 PMC1779929

[5] Levitt M (1976) A simplified representation of protein conformations for rapid simulation of protein folding. Journal of Molecular Biology 104: 59–107. 957439 10.1016/0022-2836(76)90004-8

[6] Skolnick J, Kolinski A, Ortiz AR (1997) MONSSTER: a method for folding globular proteins with a small number of distance restraints. Journal of Molecular Biology 265: 217–241. 9020984 10.1006/jmbi.1996.0720

[7] Liwo A, Oldziej S, Pincus M, Wawak J, Rackovsky S, et al (1997) A united-residue force field for off-lattice protein-structure simulations. I. Functional forms and parameters of long-range sidechain interaction potentials from protein crystal data. Journal of Computational Chemistry 18: 849–873.

[8] Zhang Y, Kolinski A, Skolnick J (2003) TOUCHSTONE II: a new approach to ab initio protein structure prediction. Biophysical Journal 85: 1145–1164. 12885659 10.1016/S0006-3495(03)74551-2 PMC1303233

[9] Kolinski A (2004) Protein modeling and structure prediction with a reduced representation. Acta Biochmica Polonica 51: 349–371. 15218533

[10] Ding F, Buldyrev SV, Dokholyan NV (2005) Folding Trp-cage to NMR resolution native structure using a coarse-grained protein model. Biophysical Journal 88: 147–155. 15533926 10.1529/biophysj.104.046375 PMC1304993

[11] MacDonald JT, Maksimiak K, Sadowski MI, Taylor WR (2010) De novo backbone scaffolds for protein design. Proteins 78: 1311–1325. 20017215 10.1002/prot.22651 PMC2841848

[12] Jefferys BR, Kelley LA, Sternberg MJE (2010) Protein folding requires crowd control in a simulated cell. Journal of Molecular Biology 397: 1329–1338. 20149797 10.1016/j.jmb.2010.01.074 PMC2891488

[13] Coluzza I (2011) A coarse-grained approach to protein design: learning from design to understand folding. PloS ONE 6: e20853. 21747930 10.1371/journal.pone.0020853 PMC3128589

[14] Marrink SJ, Risselada HJ, Yefimov S, Tieleman DP, de Vries AH (2007) The MARTINI force field: coarse grained model for biomolecular simulations. The Journal of Physical Chemistry B 111: 7812–7824. 17569554 10.1021/jp071097f

[15] Monticelli L, Kandasamy SK, Periole X, Larson RG, Tieleman DP, et al (2008) The MARTINI Coarse-Grained Force Field: Extension to Proteins. Journal of Chemical Theory and Computation 4: 819–834. 26621095 10.1021/ct700324x

[16] Gopal SM, Mukherjee S, Cheng YM, Feig M (2010) PRIMO/PRIMONA: a coarse-grained model for proteins and nucleic acids that preserves near-atomistic accuracy. Proteins 78: 1266–1281. 19967787 10.1002/prot.22645 PMC2822037

[17] Feig M, Gopal SM, Vadivel K, Stumpff-Kane A (2011) Conformational Sampling in Structure Prediction and Refinement with Atomistic and Coarse-Grained Models. In: Kolinski A, editor, Multiscale Approaches to Protein Modeling, New York, NY: Springer New York, chapter 4: 85–109.

[18] Taketomi H, Ueda Y, Gō N (1975) Studies on protein folding, unfolding and fluctuations by computer simulation. International Journal of Peptide and Protein Research 7: 445–459. 1201909

[19] Dill KA, Bromberg S, Yue K, Fiebig KM, Yee DP, et al (1995) Principles of protein folding-a perspective from simple exact models. Protein Science 4: 561–602. 7613459 10.1002/pro.5560040401 PMC2143098

[20] Simons KT, Kooperberg C, Huang E, Baker D (1997) Assembly of protein tertiary structures from fragments with similar local sequences using simulated annealing and Bayesian scoring functions. Journal of Molecular Biology 268: 209–225. 9149153 10.1006/jmbi.1997.0959

[21] Jones DT (1997) Successful ab initio prediction of the tertiary structure of NK-lysin using multiple sequences and recognized supersecondary structural motifs. Proteins Suppl 1: 185–191. 10.1002/(sici)1097-0134(1997)1+<185::aid-prot24>3.3.co;2-t 9485510

[22] Xu D, Zhang Y (2012) Ab initio protein structure assembly using continuous structure fragments and optimized knowledge-based force field. Proteins 80: 1715–1735. 22411565 10.1002/prot.24065 PMC3370074

[23] Laio A, Parrinello M (2002) Escaping free-energy minima. Proceedings of the National Academy of Sciences of the United States of America 99: 12562–12566. 12271136 10.1073/pnas.202427399 PMC130499

[24] Torrie G, Valleau J (1977) Nonphysical sampling distributions in Monte Carlo free-energy estimation: Umbrella sampling. Journal of Computational Physics 23: 187–199.

[25] Chikenji G, Fujitsuka Y, Takada S (2003) A reversible fragment assembly method for de novo protein structure prediction. The Journal of Chemical Physics 119: 6895.

[26] Holm L, Sander C (1991) Application to model building and detection of co-ordinate errors. Journal of Molecular Biology 218: 183–194. 2002501 10.1016/0022-2836(91)90883-8

[27] Milik M, Kolinski A, Skolnick J (1997) Algorithm for rapid reconstruction of protein backbone from alpha carbon coordinates. Journal of Computational Chemistry 18: 80–85.

[28] Adcock SA (2004) Peptide backbone reconstruction using dead-end elimination and a knowledgebased forcefield. Journal of Computational Chemistry 25: 16–27. 14634990 10.1002/jcc.10314

[29] Gront D, Kmiecik S, Kolinski A (2007) Backbone building from quadrilaterals: A fast and accurate algorithm for protein backbone reconstruction from alpha carbon coordinates. Journal of Computational Chemistry 28: 1593–1597. 17342707 10.1002/jcc.20624

[30] Dunbrack R (2002) Rotamer Libraries in the 21 st Century. Current Opinion in Structural Biology 12: 431–440. 12163064 10.1016/s0959-440x(02)00344-5

[31] Krivov GG, Shapovalov MV, Dunbrack RL (2009) Improved prediction of protein side-chain conformations with SCWRL4. Proteins 77: 778–795. 19603484 10.1002/prot.22488 PMC2885146

[32] Feig M (2008) Is alanine dipeptide a good model for representing the torsional preferences of protein backbones? Journal of Chemical Theory and Computation 4: 1555–1564. 26621437 10.1021/ct800153n

[33] Best R, Zhu X, Shim J (2012) Optimization of the additive CHARMM all-atom protein force field targeting improved sampling of the backbone *φ, ψ*, and side-chain *χ*1 and *χ*2 dihedral angles. Journal of Chemical Theory and Computation 8: 3257–3273. 23341755 10.1021/ct300400x PMC3549273

[34] Baker D, Sali A (2001) Protein structure prediction and structural genomics. Science 294: 93–96. 11588250 10.1126/science.1065659

[35] Kelley LA, Sternberg MJE (2009) Protein structure prediction on the Web: a case study using the Phyre server. Nature Protocols 4: 363–371. 19247286 10.1038/nprot.2009.2

[36] Kuhlman B, O’Neill JW, Kim DE, Zhang KY, Baker D (2002) Accurate computer-based design of a new backbone conformation in the second turn of protein L. Journal of Molecular Biology. 315: 471–477. 10.1006/jmbi.2001.5229 11786026

[37] Hu X, Wang H, Ke H, Kuhlman B (2007) High-resolution design of a protein loop. Proceedings of the National Academy of Sciences of the United States of America 104: 17668–17673. 17971437 10.1073/pnas.0707977104 PMC2077077

[38] Eiben CB, Siegel JB, Bale JB, Cooper S, Khatib F, et al (2012) Increased Diels-Alderase activity through backbone remodeling guided by Foldit players. Nature Biotechnology 30: 190–192. 10.1038/nbt.2109 PMC3566767 22267011

[39] Fiser A, Do RKG, Sali A (2000) Modeling of loops in protein structures. Protein Science 9: 1753–1773. 11045621 10.1110/ps.9.9.1753 PMC2144714

[40] Xiang Z, Soto CS, Honig B (2002) Evaluating conformational free energies: the colony energy and its application to the problem of loop prediction. Proceedings of the National Academy of Sciences of the United States of America 99: 7432–7437. 12032300 10.1073/pnas.102179699 PMC124248

[41] DePristo MA, de Bakker PIW, Lovell SC, Blundell TL (2003) Ab initio construction of polypeptide fragments: Effcient generation of accurate, representative ensembles. Proteins 51: 41–55. 12596262 10.1002/prot.10285

[42] Jacobson MP, Pincus DL, Rapp CS, Day TJF, Honig B, et al (2004) A hierarchical approach to all-atom protein loop prediction. Proteins 55: 351–367. 15048827 10.1002/prot.10613

[43] Rohl CA, Strauss CEM, Chivian D, Baker D (2004) Modeling structurally variable regions in homologous proteins with Rosetta. Proteins 55: 656–677. 15103629 10.1002/prot.10629

[44] Mönnigmann M, Floudas CA (2005) Protein loop structure prediction with exible stem geometries. Proteins 61: 748–762. 16222670 10.1002/prot.20669

[45] Zhu K, Pincus D, Zhao S, Friesner RA (2006) Long loop prediction using the protein local optimization program. Proteins 65: 438–452. 16927380 10.1002/prot.21040

[46] Soto C, Fasnacht M, Zhu J, Forrest L, Honig B (2007) Loop modeling: sampling, filtering, and scoring. Proteins 70: 834–843. 10.1002/prot.21612 PMC2553011 17729286

[47] Peng HP, Yang AS (2007) Modeling protein loops with knowledge-based prediction of sequencestructure alignment. Bioinformatics 23: 2836–2842. 17827204 10.1093/bioinformatics/btm456

[48] Sellers BD, Zhu K, Zhao S, Friesner RA, Jacobson MP (2008) Toward better refinement of comparative models: predicting loops in inexact environments. Proteins 72: 959–971. 18300241 10.1002/prot.21990 PMC2764870

[49] Mandell DJ, Coutsias EA, Kortemme T (2009) Sub-angstrom accuracy in protein loop reconstruction by robotics-inspired conformational sampling. Nature Methods 6: 551–552. 19644455 10.1038/nmeth0809-551 PMC2847683

[50] Choi Y, Deane CM (2010) FREAD revisited: Accurate loop structure prediction using a database search algorithm. Proteins 78: 1431–1440. 20034110 10.1002/prot.22658

[51] Jamroz M, Kolinski A (2010) Modeling of loops in proteins: a multi-method approach. BMC Structural Biology 10: 5. 20149252 10.1186/1472-6807-10-5 PMC2837870

[52] Lee J, Lee D, Park H, Coutsias EA, Seok C (2010) Protein loop modeling by using fragment assembly and analytical loop closure. Proteins 78: 3428–3436. 20872556 10.1002/prot.22849 PMC2976774

[53] Marks DS, Colwell LJ, Sheridan R, Hopf TA, Pagnani A, et al (2011) Protein 3D structure computed from evolutionary sequence variation. PloS ONE 6: e28766. 22163331 10.1371/journal.pone.0028766 PMC3233603

[54] Kim DE, Blum B, Bradley P, Baker D (2009) Sampling bottlenecks in de novo protein structure prediction. Journal of Molecular Biology 393: 249–260. 19646450 10.1016/j.jmb.2009.07.063 PMC2760740

[55] Pandini A, Fornili A, Kleinjung J (2010) Structural alphabets derived from attractors in conformational space. BMC Bioinformatics 11: 97. 20170534 10.1186/1471-2105-11-97 PMC2838871

[56] Weiner SJ, Kollman PA, Case DA, Singh UC, Ghio C, et al (1984) A new force field for molecular mechanical simulation of nucleic acids and proteins. Journal of the American Chemical Society 106: 765–784.

[57] Kortemme T, Morozov AV, Baker D (2003) An orientation-dependent hydrogen bonding potential improves prediction of specificity and structure for proteins and protein-protein complexes. Journal of Molecular Biology 326: 1239–1259. 12589766 10.1016/s0022-2836(03)00021-4

[58] Chaudhury S, Lyskov S, Gray JJ (2010) Pyrosetta: a script-based interface for implementing molecular modeling algorithms using rosetta. Bioinformatics 26: 689–691. 20061306 10.1093/bioinformatics/btq007 PMC2828115

[59] Khatib F, Cooper S, Tyka MD, Xu K, Makedon I, et al (2011) Algorithm discovery by protein folding game players. Proceedings of the National Academy of Sciences of the United States of America 108: 18949–18953. 22065763 10.1073/pnas.1115898108 PMC3223433

[60] Altschul SF, Madden TL, Schäffer AA, Zhang J, Zhang Z, et al (1997) Gapped blast and psi-blast: a new generation of protein database search programs. Nucleic Acids Research 25: 3389–3402. 9254694 10.1093/nar/25.17.3389 PMC146917

[61] Wang G, Dunbrack RL (2003) PISCES: a protein sequence culling server. Bioinformatics 19: 1589–1591. 12912846 10.1093/bioinformatics/btg224

[62] Ortiz A, Strauss C, Olmea O (2002) MAMMOTH (matching molecular models obtained from theory): an automated method for model comparison. Protein Science 11: 2606–2621. 12381844 10.1110/ps.0215902 PMC2373724

[63] Frishman D, Argos P (1995) Knowledge-based protein secondary structure assignment. Proteins 23: 566–579. 8749853 10.1002/prot.340230412

[64] de Bakker PIW, DePristo Ma, Burke DF, Blundell TL (2003) Ab initio construction of polypeptide fragments: Accuracy of loop decoy discrimination by an all-atom statistical potential and the AMBER force field with the Generalized Born solvation model. Proteins 51: 21–40. 12596261 10.1002/prot.10235

