# Supplementary material for: Validating a Coarse-Grained Potential Energy Function through Protein Loop Modelling
Source: PLoS One. 2013 Jun 18;8(6):e65770. doi: 10.1371/journal.pone.0065770 (PMC3688807; doi:10.1371/journal.pone.0065770)
Supplement: File S1 — Supporting figures and tables. (PDF) [file pone.0065770.s001.pdf]

Supplementary Information: Validating a coarse-grained potential energy function through protein loop modelling

James T. MacDonald[1] , Lawrence A. Kelley, Paul S. Freemont

April 23, 2013

[1]j.macdonald@imperial.ac.uk Division of Molecular Biosciences, Imperial College London, SW7 2AZ

	backbone RMSD (Å)			
residues	RAPPER	PD2	PD2_rmin	FALCm4
2	0.31			
3	0.34	0.32	0.23	
4	0.43	0.27	**0.16**	0.33
5	0.53	0.59	0.46	0.44
6	0.69	0.83	0.66	0.47
7	0.78	1.22	0.91	**0.58**
8	1.11	1.41	1.06	0.84
9	1.29	1.41	1.07	**0.95**
10	1.67	1.71	**1.41**	1.45
11	1.99	2.30	2.02	**1.47**
12	2.21	2.95	2.34	**1.74**

Table S1: Best RMSD-G from Figure 1.

	backbone RMSD (Å)			
residues	RAPPER	PD2	PD2_rmin	FALCm4
2	0.95			
3	1.30	0.51	0.43	
4	1.65	0.89	0.68	0.92
5	2.27	1.91	1.66	1.63
6	3.06	2.97	2.66	2.34
7	3.79	3.65	3.36	**2.74**
8	4.16	4.09	3.79	3.69
9	5.00	4.39	4.18	**4.21**
10	5.66	5.02	4.70	5.07
11	6.71	6.20	5.99	**5.76**
12	6.96	7.19	6.86	6.31

Table S2: Ensemble RMSD-G from Figure 1.

residues	RAPPER	PD2	PD2_rmin
2	0.89		
3	0.80	0.97	0.97
4	0.72	0.92	0.96
5	0.45	0.60	0.62
6	0.24	0.39	0.47
7	0.14	0.32	0.37
8	0.12	0.16	0.23
9	0.04	0.11	0.16
10	0.02	0.05	0.09
11	0.01	0.01	0.02
12	0.01	0.00	0.01

Table S3: Ensemble fraction below 2 Å RMSD-G from Figure 1.

residues	RAPPER	PD2	PD2_rmin
2	0.67		
3	0.31	0.89	0.91
4	0.18	0.69	0.78
5	0.11	0.37	0.45
6	0.03	0.16	0.26
7	0.02	0.09	0.18
8	0.01	0.02	0.07
9	0.00	0.02	0.06
10	0.00	0.00	0.01
11	0.00	0.00	0.00
12	0.00	0.00	0.00

Table S4: Ensemble fraction below 1 Å RMSD-G from Figure 1.

			ensemble % below 1 Å RMSD				ensemble % below 2 Å RMSD			
PDB	residues	length	PD2	PD2_rmin	control	control_rmin	PD2	PD2_rmin	control	control_rmin
3zbd	76A-83A	8	0.0	0.0	0.0	0.0	1.1	18.6	0.1	0.8
4f55	202A-209A	8	0.2	3.5	0.0	0.0	12.3	16.3	0.1	0.2
4f55	222A-230A	9	0.0	0.0	0.0	0.0	0.0	0.0	0.0	0.0
4fc9	553A-563A	11	0.0	0.0	0.0	0.0	0.0	0.0	0.0	0.1
4fch	186A-194A	9	0.0	0.0	0.0	0.0	0.4	0.7	0.0	0.0
4fch	303A-313A	11	0.3	1.3	0.0	0.0	6.4	10.1	0.0	0.2
4fch	350A-357A	8	0.0	0.0	0.0	0.0	0.8	4.6	1.8	6.8

Table S5: Proportion of new structures loop test set below 1 Åand 2 Å

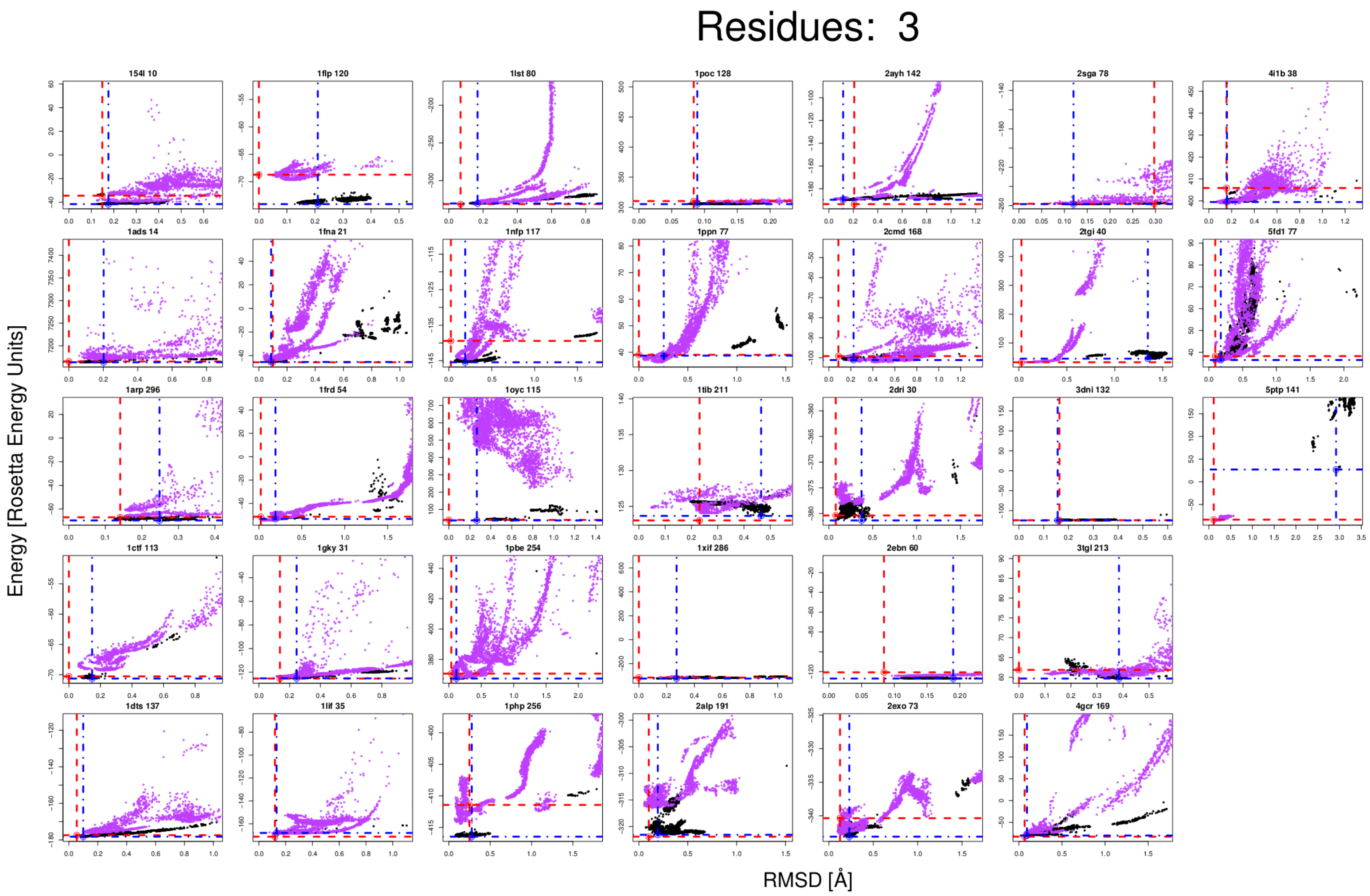

Figure S1: Loop backbone RMSD-G vs Rosetta energy. The red point indicates the energy minimised crystal structure loop and the blue point indicates the lowest energy PD2_rmin decoy loop conformation. The black points correspond to PD2_rmin loop decoys while the purple points correspond to the control_rmin loop decoys.

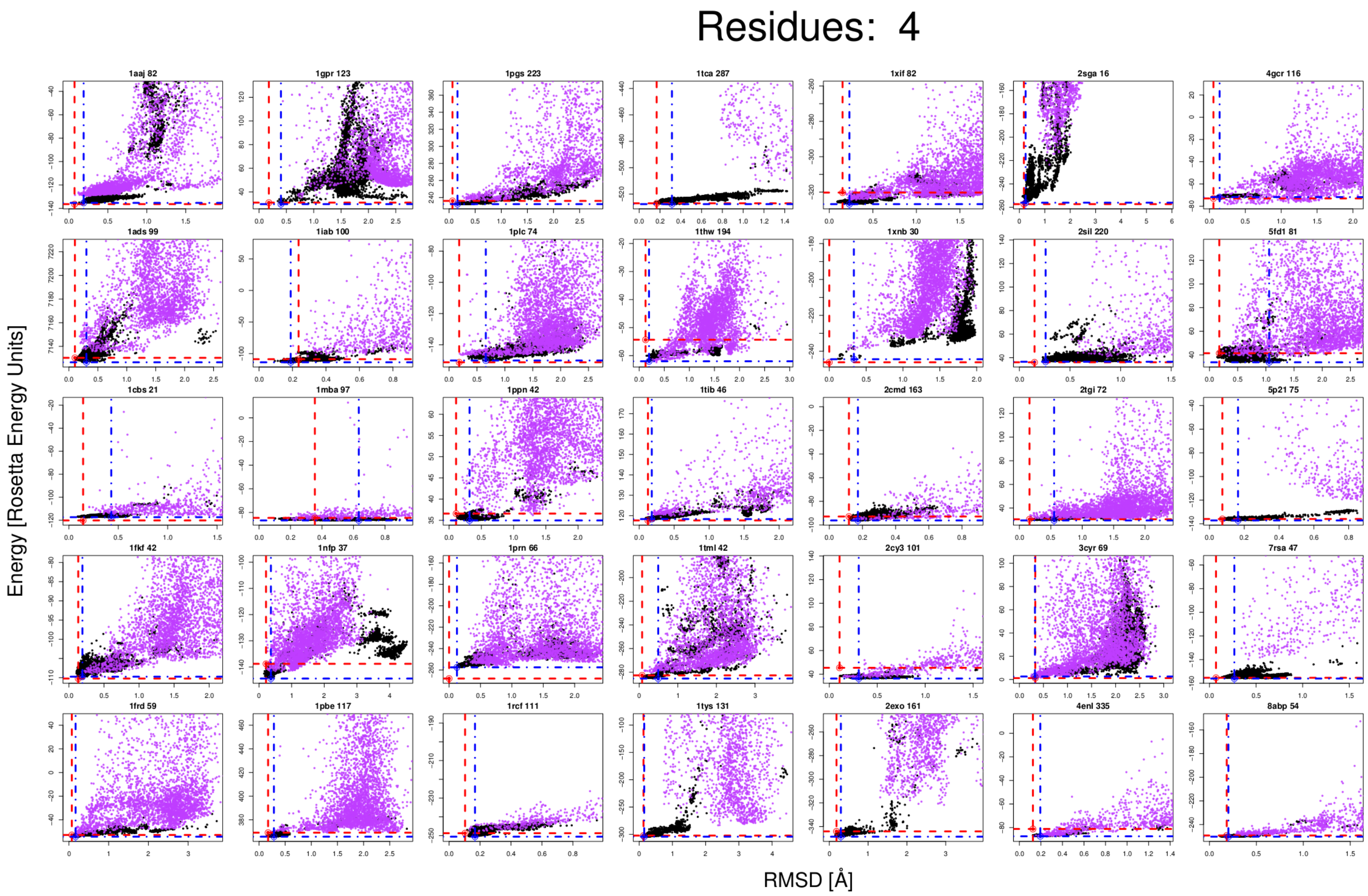

Figure S2: Loop backbone RMSD-G vs Rosetta energy. The red point indicates the energy minimised crystal structure loop and the blue point indicates the lowest energy PD2_rmin decoy loop conformation. The black points correspond to PD2_rmin loop decoys while the purple points correspond to the control_rmin loop decoys.

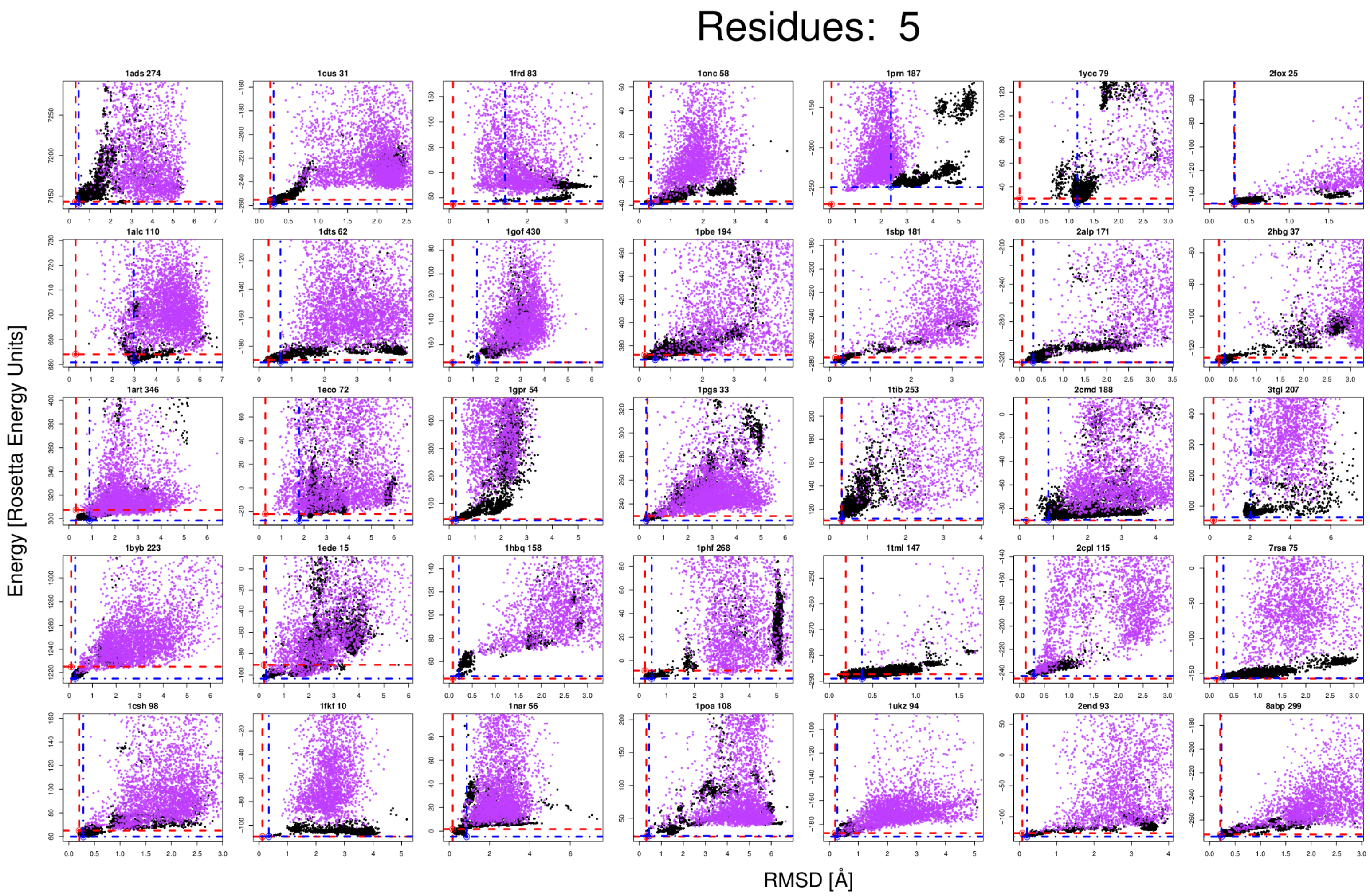

Figure S3: Loop backbone RMSD-G vs Rosetta energy. The red point indicates the energy minimised crystal structure loop and the blue point indicates the lowest energy PD2_rmin decoy loop conformation. The black points correspond to PD2_rmin loop decoys while the purple points correspond to the control_rmin loop decoys.

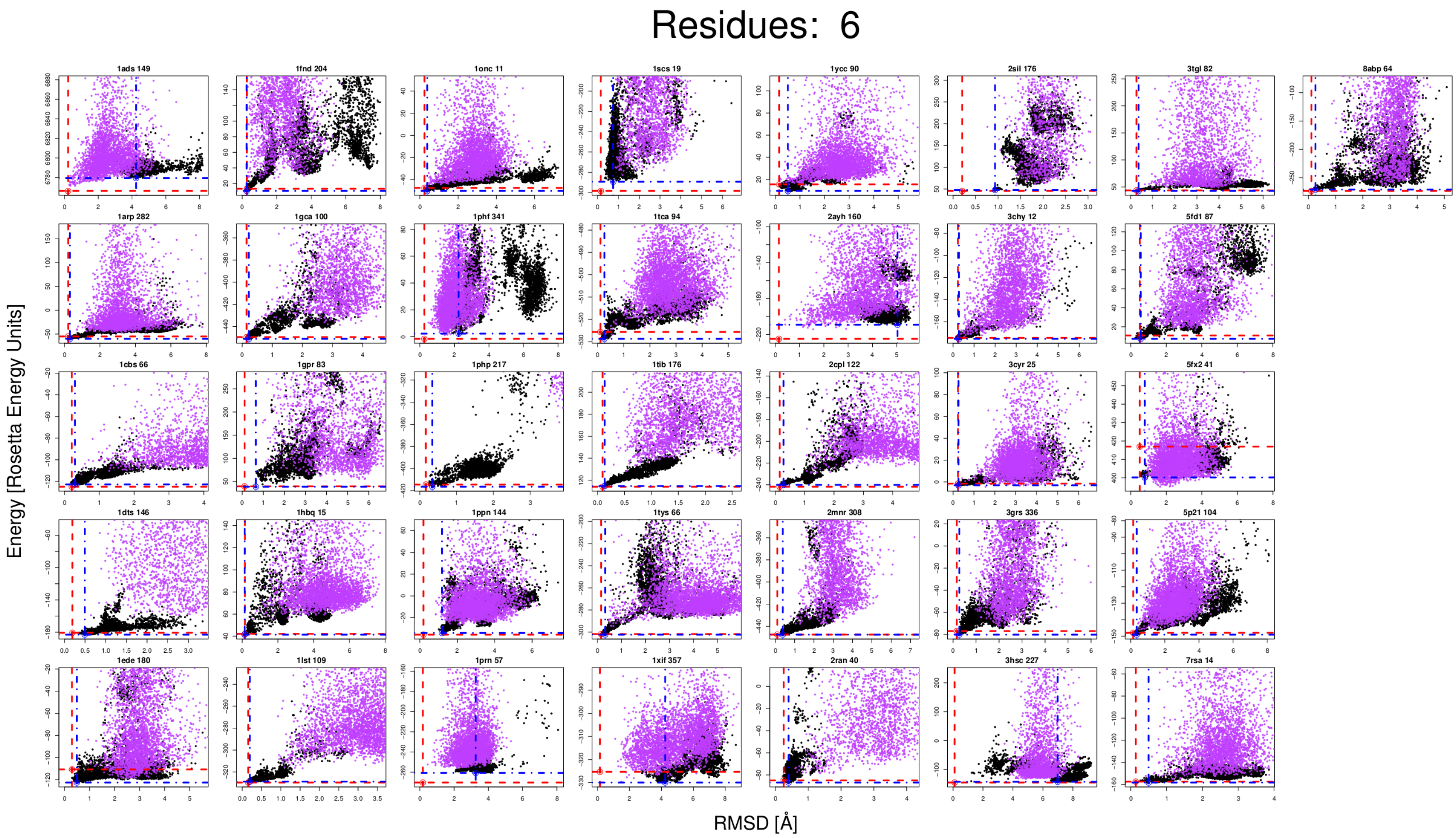

Figure S4: Loop backbone RMSD-G vs Rosetta energy. The red point indicates the energy minimised crystal structure loop and the blue point indicates the lowest energy PD2_rmin decoy loop conformation. The black points correspond to PD2_rmin loop decoys while the purple points correspond to the control_rmin loop decoys.

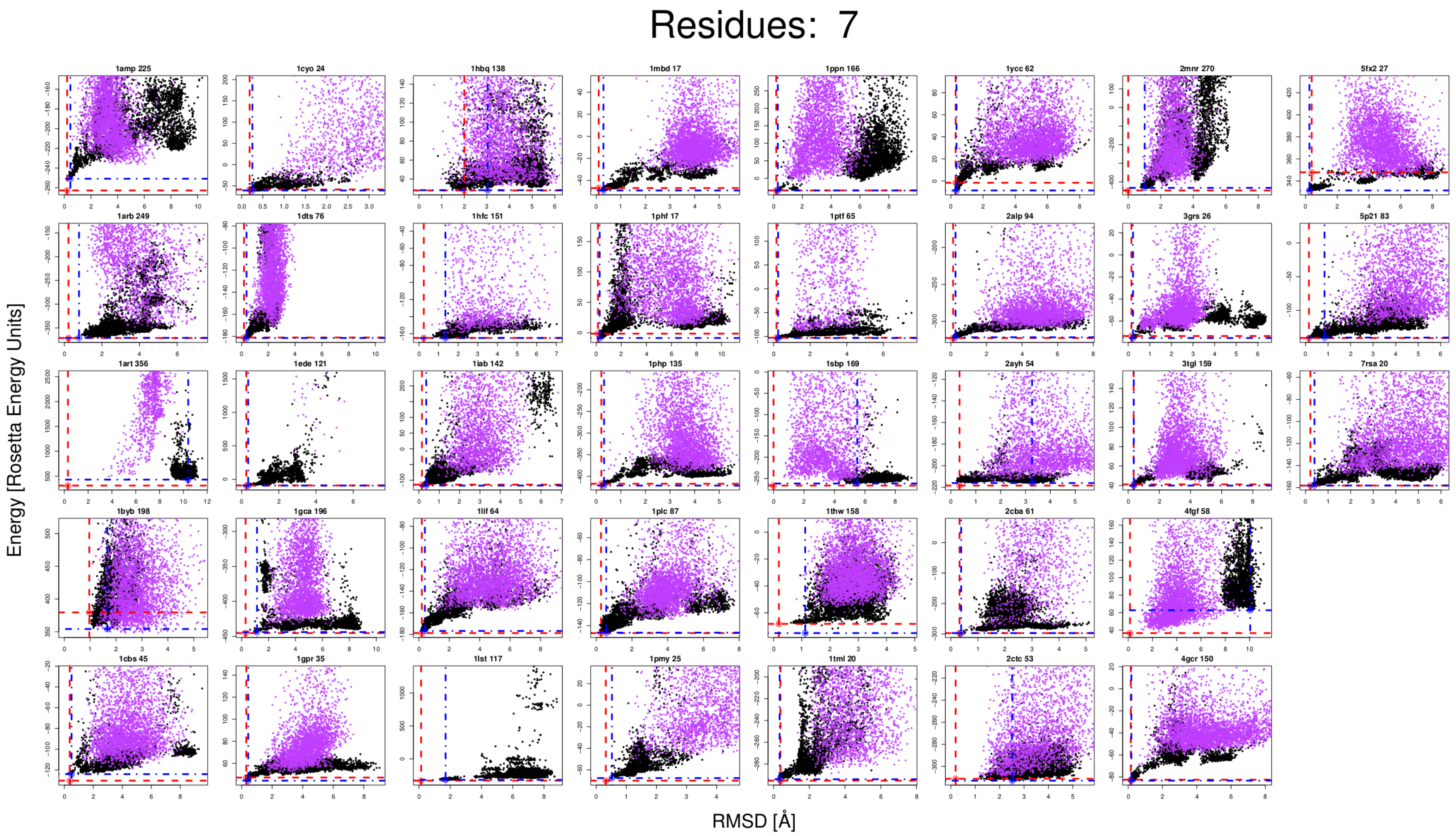

Figure S5: Loop backbone RMSD-G vs Rosetta energy. The red point indicates the energy minimised crystal structure loop and the blue point indicates the lowest energy PD2_rmin decoy loop conformation. The black points correspond to PD2_rmin loop decoys while the purple points correspond to the control_rmin loop decoys.

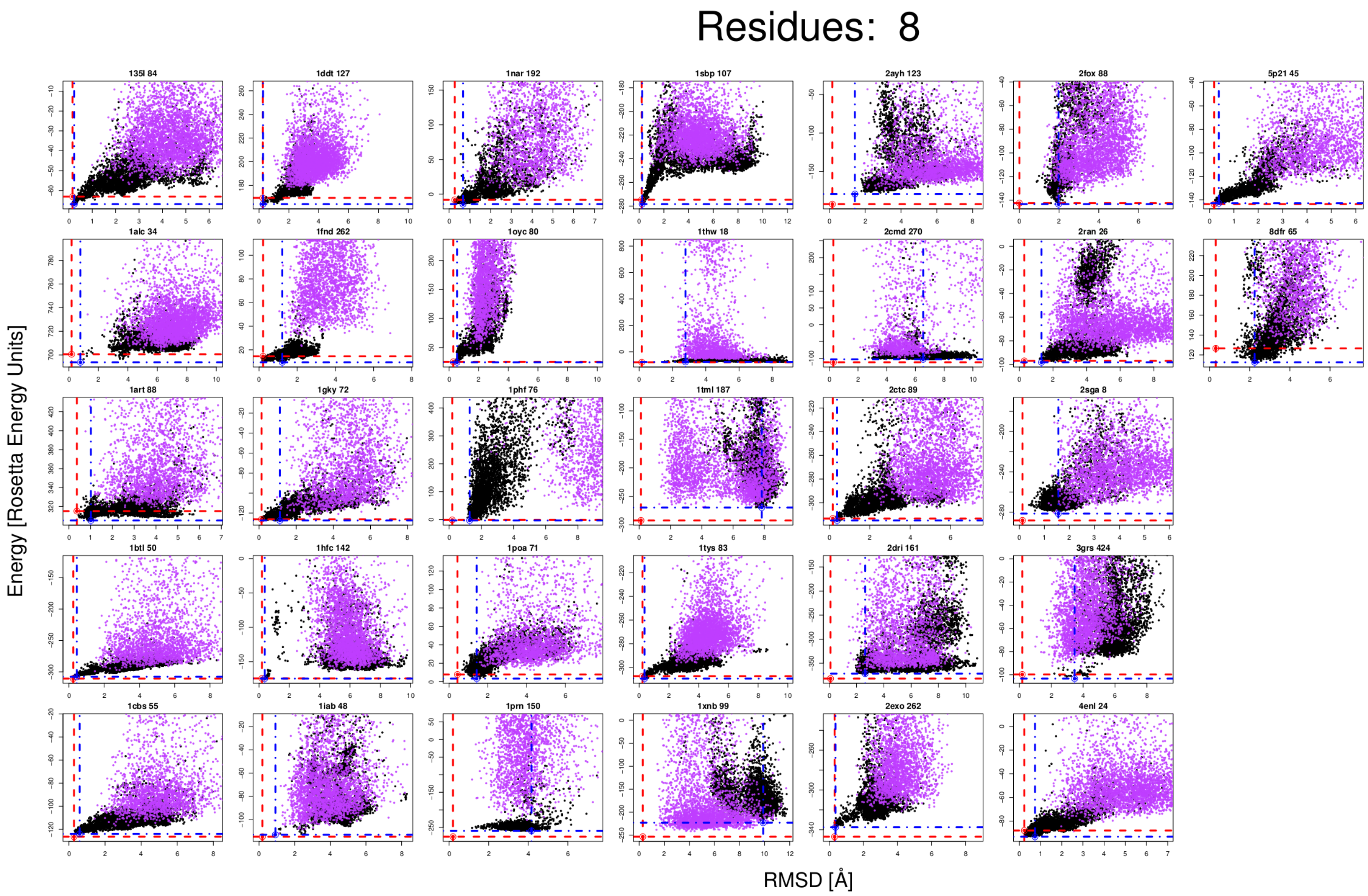

Figure S6: Loop backbone RMSD-G vs Rosetta energy. The red point indicates the energy minimised crystal structure loop and the blue point indicates the lowest energy PD2_rmin decoy loop conformation. The black points correspond to PD2_rmin loop decoys while the purple points correspond to the control_rmin loop decoys.

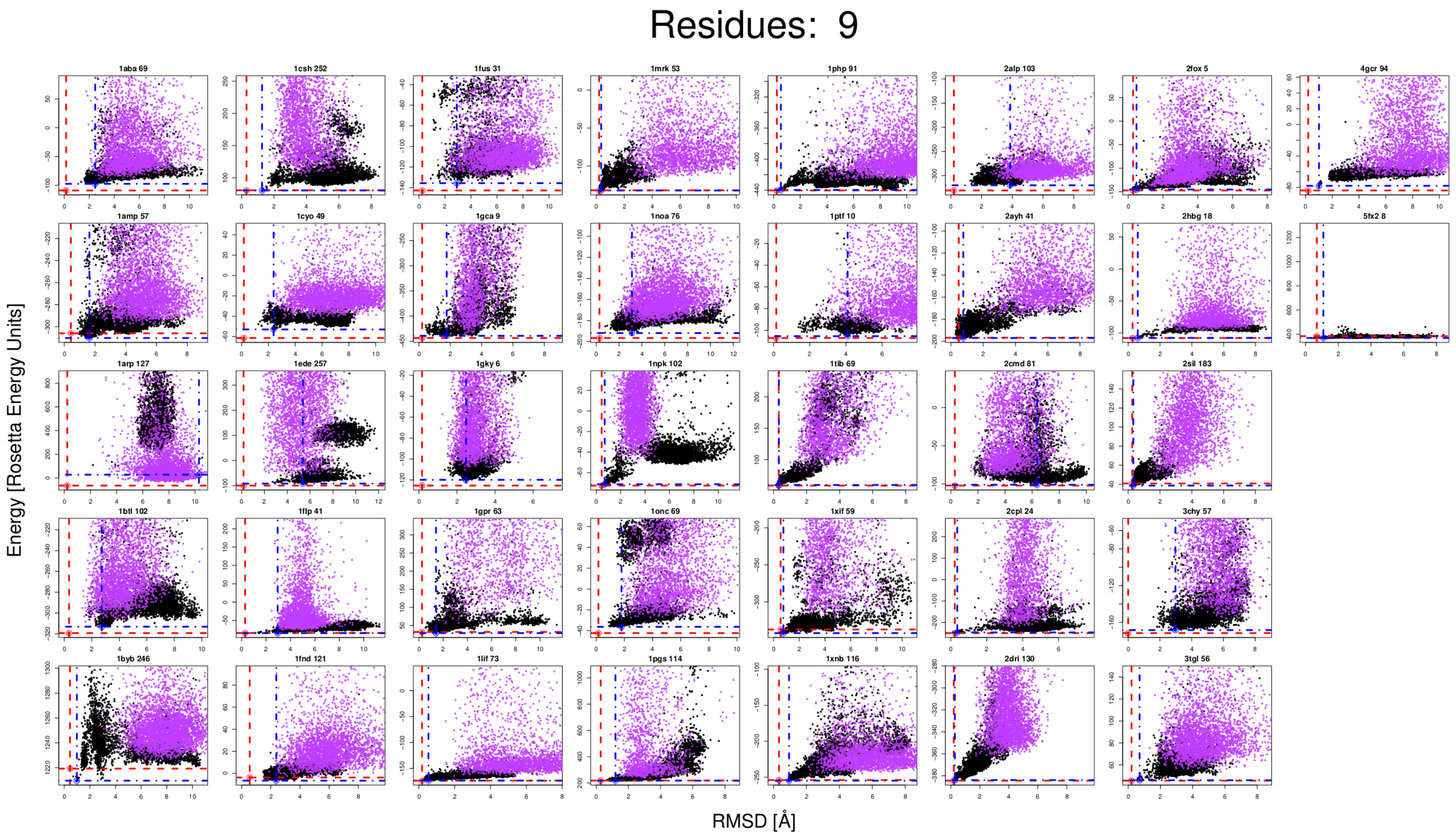

Figure S7: Loop backbone RMSD-G vs Rosetta energy. The red point indicates the energy minimised crystal structure loop and the blue point indicates the lowest energy PD2_rmin decoy loop conformation. The black points correspond to PD2_rmin loop decoys while the purple points correspond to the control_rmin loop decoys.

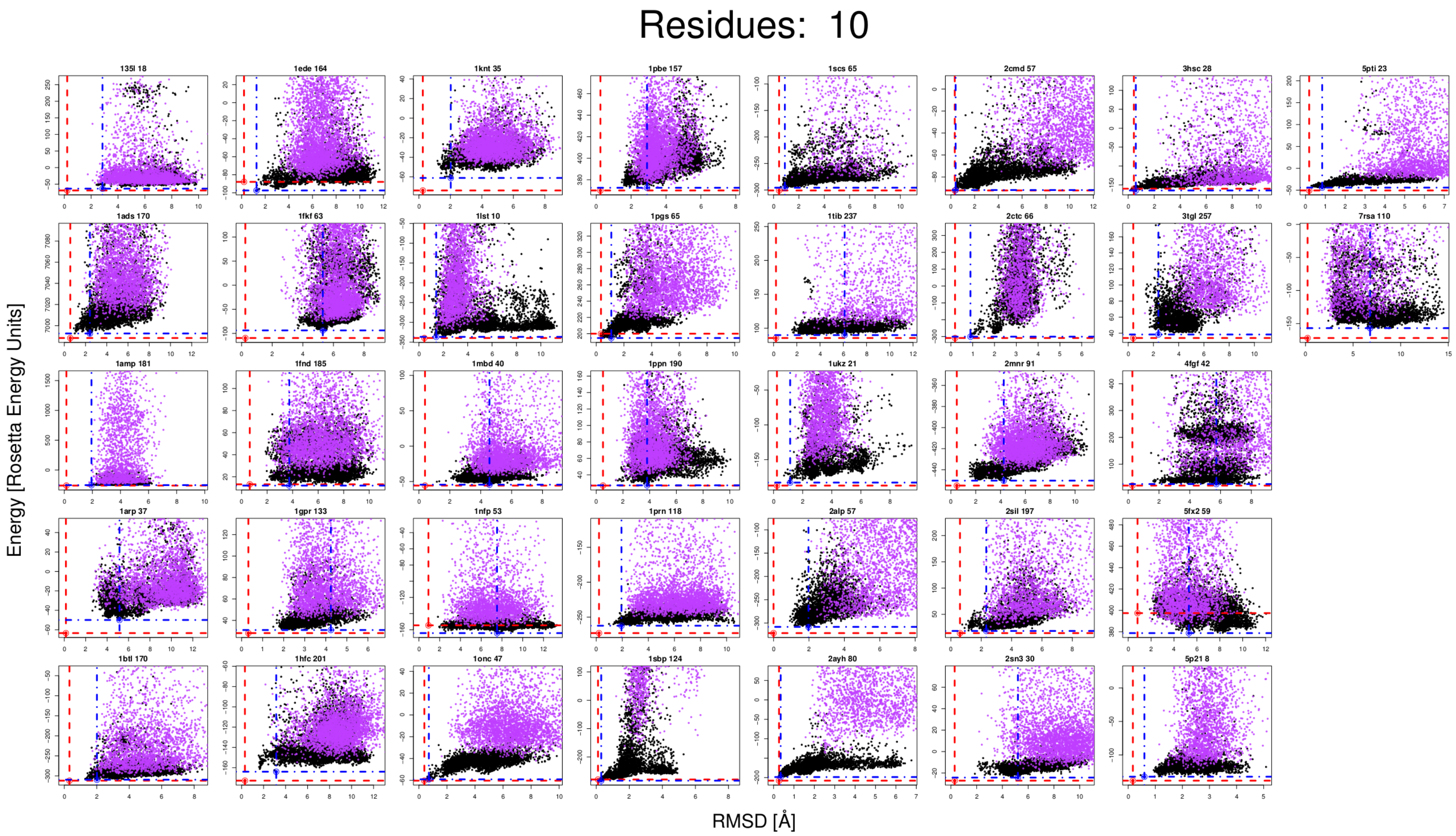

Figure S8: Loop backbone RMSD-G vs Rosetta energy. The red point indicates the energy minimised crystal structure loop and the blue point indicates the lowest energy PD2_rmin decoy loop conformation. The black points correspond to PD2_rmin loop decoys while the purple points correspond to the control_rmin loop decoys.

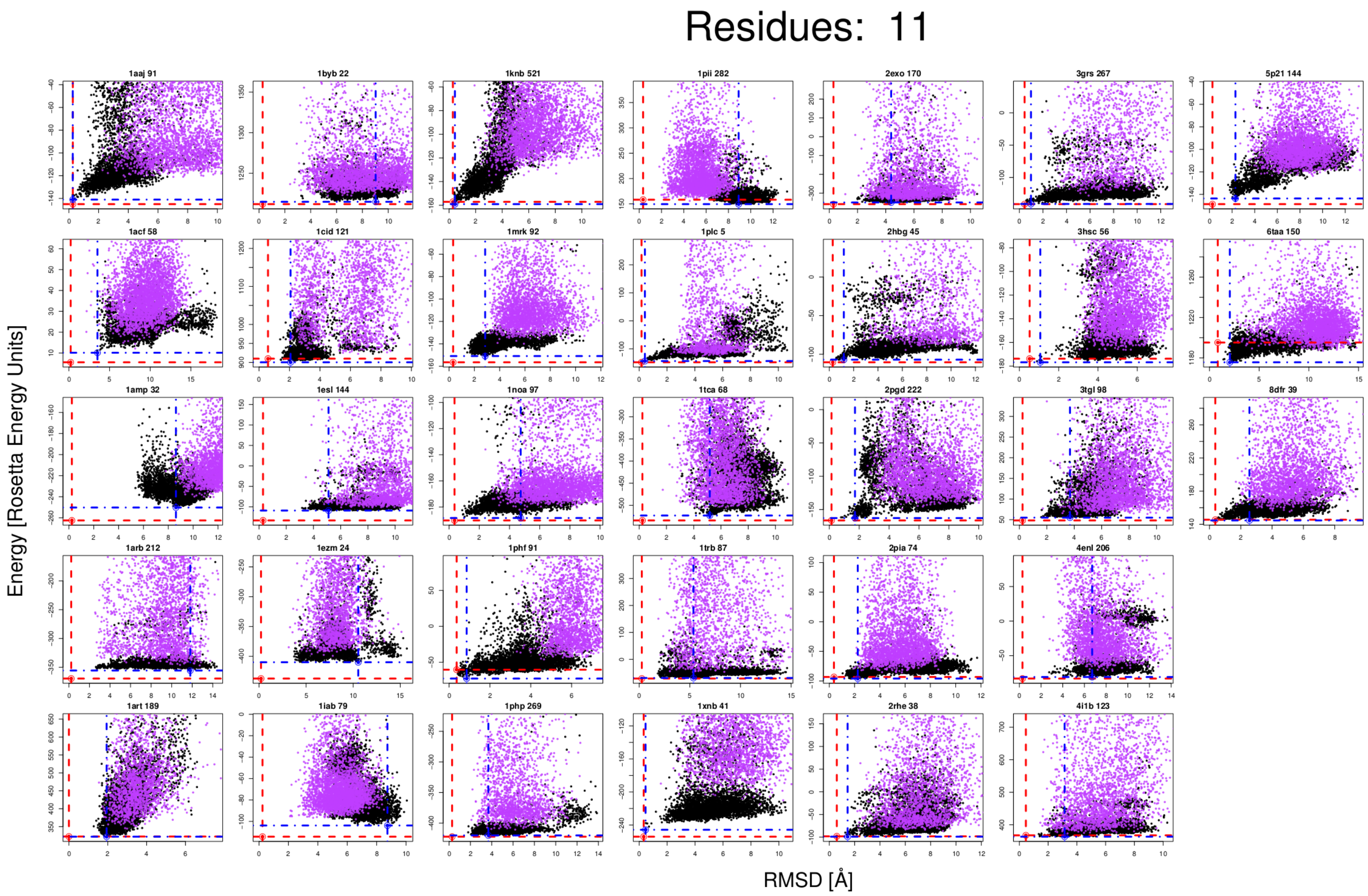

Figure S9: Loop backbone RMSD-G vs Rosetta energy. The red point indicates the energy minimised crystal structure loop and the blue point indicates the lowest energy PD2_rmin decoy loop conformation. The black points correspond to PD2_rmin loop decoys while the purple points correspond to the control_rmin loop decoys.

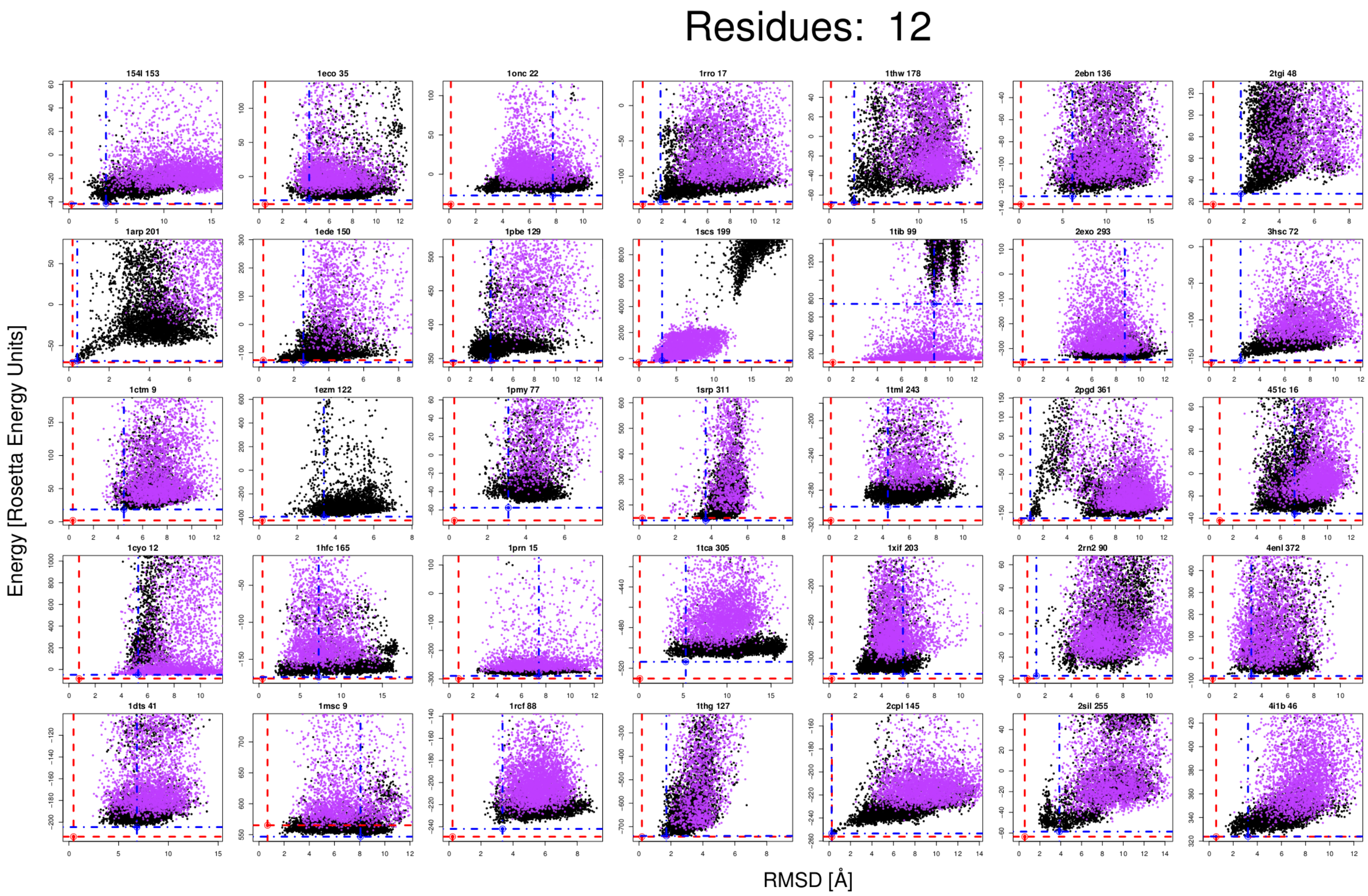

Figure S10: Loop backbone RMSD-G vs Rosetta energy. The red point indicates the energy minimised crystal structure loop and the blue point indicates the lowest energy PD2_rmin decoy loop conformation. The black points correspond to PD2_rmin loop decoys while the purple points correspond to the control_rmin loop decoys.
